# In focus: data management and data analysis in microscopy

**DOI:** 10.1007/s00418-023-02226-0

**Published:** 2023-08-30

**Authors:** Ben N. G. Giepmans, Douglas J. Taatjes, Katherine J. Wolstencroft

**Affiliations:** 1 Department of Biomedical Sciences of Cells and Systems, University Medical Center Groningen, University of Groningen, Groningen, The Netherlands; 2 Department of Pathology and Laboratory Medicine, Larner College of Medicine, University of Vermont, Burlington, VT 05405 USA; 3 The Leiden Institute of Advanced Computer Science (LIACS), Leiden University, Leiden, The Netherlands

## Summary

Technological advances in spatiotemporal, volume, and high-resolution imaging, as well as automated and digital microscopy, have resulted in an avalanche of (multimodal) imaging data. Data management and analysis are becoming bottlenecks that require increased automation and novel approaches. Additionally, reusing such data and integrating it with other life science disciplines provide new opportunities for research, but require greater standardization and data sharing. Making bioimaging data FAIR (Findable, Accessible, Interoperable, and Reusable) is becoming a central concern for global bioimaging initiatives. In this special issue, we survey the landscape of large volume, multimodal bioimaging data management and analysis techniques, exploring the challenges and opportunities.

In this special issue of *Histochemistry and Cell Biology* we address the very timely topic of data management and data analysis in microscopy imaging. With national funding agencies increasingly requiring data management plans in grant proposals, the issue of data management for images has been brought to the forefront. The data avalanche in microscopy was accelerated by both the change from analog to digital cameras and innovations in multidimensional image scanning procedures. With these developments in automated image acquisition, microscopists are now able to generate data around the clock. Consequently, data management and data analysis have become major challenges. To put this into context, recall that only 20 years ago a 64 Mb USB storage device (costing US $100) would solve most data storage problems. Currently, most microscopy core facilities are managing up to petabytes per year; exa, zetta, yota, and bronto bytes will soon follow.

**Fig. 1:**
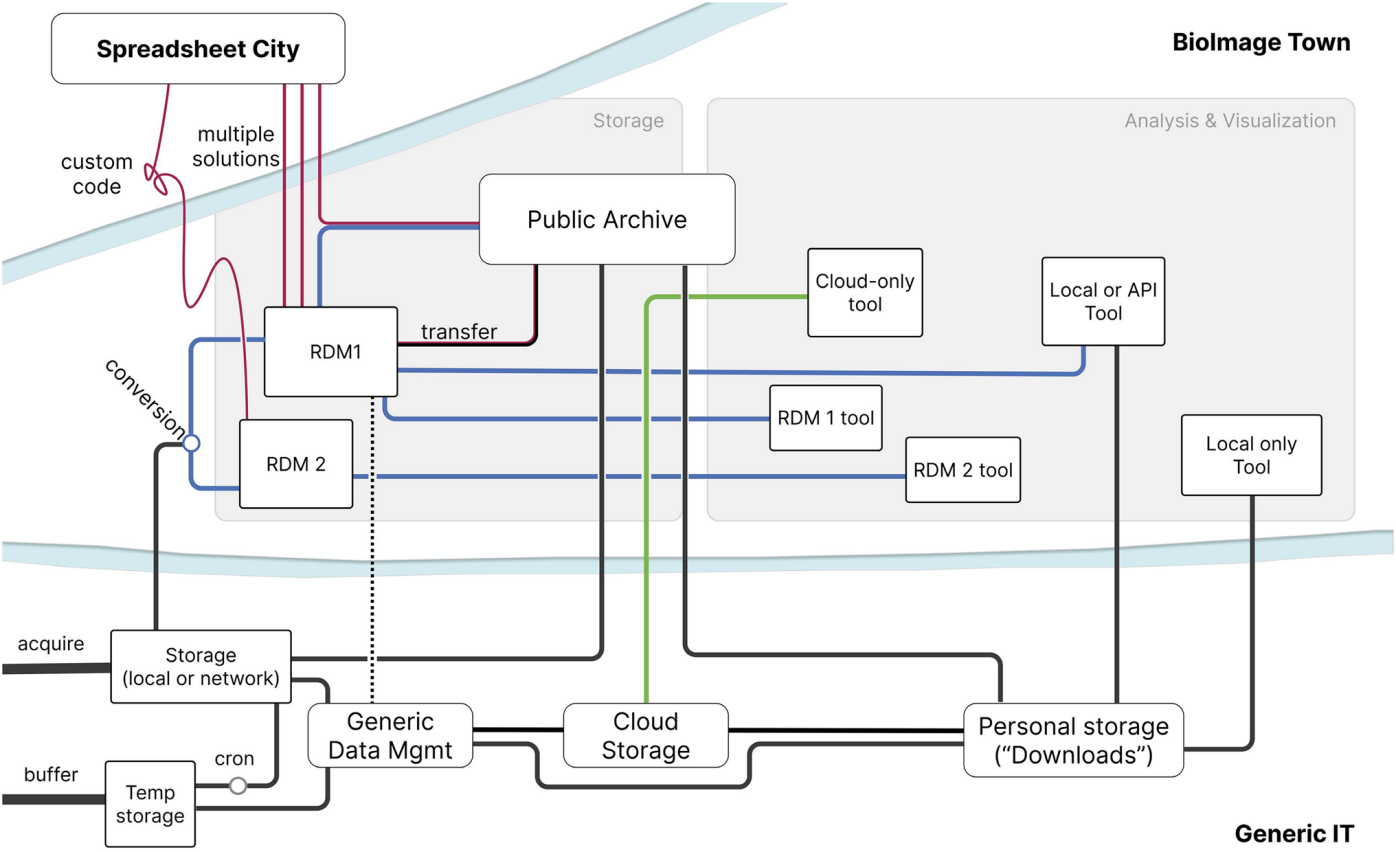
The Bioimage Town metro map. Experimental metadata (red) is integrated with acquired images (black). From here data can be analyzed, visualized, and/or shared in the public domain (blue) for further analysis and reuse (green). BioImage Town was created to integrate the different presentations of data scientists at the European Light Microscopy Initiative meeting (ELMI 2023, the Netherlands; Moore et al. 2023a, b)

The increase in data volume and heterogeneity requires new considerations and approaches for storage and sharing, and presents opportunities for innovation in image analysis methods. Collaborations between microscopists and data scientists are driving a new generation of approaches. An ecosystem of methods, analysis tools, and data resources are emerging to support microscopists. The cross-disciplinary focus on the FAIR data principles, i.e., that data should be Findable, Accessible, Interoperable, and Reusable (Wilkinson et al. 2016), underpins these developments and serves to interlink bioimaging data with omics data and other life science resources. For image analysis, growing collections of high quality, annotated imaging data can be used for training the next generation of AI algorithms. Standards in scientific workflows and integrated analysis toolsets make these developments more accessible to a wider community. Many of these developments have come from bottom-up initiatives in the bioimaging community. The Bioimage Town metro map (Fig. 1) shows how these developments could be integrated to create a connected infrastructure. Thus, in this special issue we aim to showcase the latest developments, current challenges, and future directions across Bioimage Town, for bioimaging data management and analysis.

The special issue begins with a detailed summary of the current challenges and opportunities that exist in electron microscopy (EM) by Poger et al. (2023): The data workflow is described from the perspective of core imaging facilities, with a comprehensive overview of current solutions. In many institutes, the perhaps underappreciated role that libraries play to support and assist research scientists throughout the bioimage data life cycle in digital data management is emphasized by Silkotch et al. (2023). Indeed, their field-specific knowledge and international networks are critical aspects of the support offered by library scientists in this area. Moreover, community consensus on how this should be achieved is already emerging. For instance, Euro-BioImaging, the European research infrastructure to facilitate access to advanced microscopy for scientists, present the current landscape and future perspectives on the bioimage data ecosystem (Kemmer et al. 2023). Data access via open repositories is a key component of the community strategy and the European repositories are introduced. Two of these are managed by the European Bioinformatics Institute (EBI) and their history and perspective are provided by Hartley et al. (2023). Certainly, public repositories need to store and represent data from across the myriad aspects of bioimaging and therefore must contend with the heterogeneity of data formats. The Open Microscopy Environment (OME) standards initiative leads the way in these activities. Given the increase in file sizes and the transfer to cloud-based storage and analysis, new approaches are required to represent multidimensional large datasets. In this context, the OME-Zarr file format, which is rapidly becoming the de facto standard for large datasets, is introduced in this special issue by a united team of more than 40 scientists (Moore et al. 2023a, b). Finally, this special issue concludes with a broader perspective on reusing and analyzing data. While the automation of object detection in image data is currently booming (e.g., the segment anything model; SAM; segment-anything.com), today’s best performance of complex large-scale image data annotation is generally still performed by humans. Scaling up this activity to cope with the increase in data availability therefore requires an increase in the number of people involved. Two recent successful examples of “citizen science projects” are presented (Smith et al. 2023), nicely illustrating how the public can contribute to such activities.

Technological and computational innovations are accelerating microscopy-based imaging advances, but are presenting new challenges. How do we keep pace with the ever-expanding amounts of data and knowledge, and should we keep all the data generated? What breakthroughs will be possible with the provision of FAIR data infrastructure? Can AI methods further automate image analysis and close the “annotation gap”? Key to answering these questions is the further strengthening of connections in the Bioimage Town metro map between microscopists, data scientists, and policy makers. Emphasis should therefore be on connection and interactions of these specialists, drawing on different expertise to develop a better understanding of the underlying biology we are imaging.

Many leaders in the field have contributed to this special issue. Some of the ideas are clearly aligned, others are overlapping or running more in parallel. A growing community of researchers are beginning to focus on the specific challenges of data and analysis in this field, and we foresee further alignment and community consensus in the near future as we move forwards. The result will be a FAIRer bioimaging research landscape, capable of exploiting the latest image analysis methods to accelerate discovery and biological insights.

## References

[CR1] Hartley M, Ludin A, Padwardhan A (2023). Providing open image data at scale: an EMBL-EBI perspective. Histochem Cell Biol.

[CR2] Kemmer I, Keppler A, Serrano-Solano B (2023). Building a FAIR image data ecosystem for microscopy communities. Histochem Cell Biol.

[CR3] Moore J, Basurto-Lozada D, Besson S (2023). Ome-Zarr: a cloud-optimized bioimaging file format with international community support. Histochem Cell Biol.

[CR4] Moore JM, Wolstencroft H (2023) BioImage Town (BIT) FAIR Data Metro Map [ELMI2023] (2023). Zenodo. https://zenodo.org/record/8130315.

[CR5] Poger D, Yen L, Braet F (2023). Big data in contemporary electron microscopy: challenges and opportunities in data transfer, compute and management. Histochem Cell Biol.

[CR6] Silkotch C, Garcia-Milian R, Hersey D (2023). Partnering with health sciences libraries to address challenges in bioimaging data management and sharing. Histochem Cell Biol.

[CR7] Smith P, King ONF, Pennington A (2023). Online citizen science with the Zooniverse for analysis of biological volumetric data. Histochem Cell Biol.

[CR8] Wilkinson MD, Dumontier M, Aalbersberg IJ (2016). The FAIR guiding principles for scientific data management and stewardship. Sci Data.

